# Disseminating health research to public health policy-makers and practitioners: a survey of source, message content and delivery modality preferences

**DOI:** 10.1186/s12961-023-01066-7

**Published:** 2023-11-27

**Authors:** Sam McCrabb, Alix Hall, Andrew Milat, Adrian Bauman, Rebecca Hodder, Kaitlin Mooney, Emily Webb, Courtney Barnes, Serene Yoong, Rachel Sutherland, Luke Wolfenden

**Affiliations:** 1 School of Medicine and Public Health, College of Health, Medicine and Wellbeing, University of Newcastle, Callaghan, NSW 2308 Australia; 2 Hunter Medical Research Institute, Newcastle, NSW 2305 Australia; 3 Hunter New England Population Health, Hunter New England Local Health District, Wallsend, NSW 2287 Australia; 4 School of Public Health, University of Sydney, Sydney, NSW Australia; 5 Centre for Epidemiology and Evidence, NSW Ministry of Health, Sydney, Australia; 6 Prevention Research Collaboration, Charles Perkins Centre, School of Public Health, Faculty of Medicine and Health, The University of Sydney, Sydney, NSW Australia; 7 The Australian Prevention Partnership Centre, Sydney, NSW Australia; 8 School of Health Sciences, Swinburne University of Technology, Melbourne, VIC 3122 Australia; 9 Global Nutrition and Preventive Health, Institute of Health Transformation, School of Health and Social Development, Deakin University, Burwood, Australia

**Keywords:** Dissemination, Public health, Practitioners, Implementation, Research impact

## Abstract

**Background:**

Understanding the views of policy-makers and practitioners regarding how best to communicate research evidence is important to support research use in their decision-making.

**Aim:**

To quantify and describe public health policy-makers and practitioners’ views regarding the source, content and form of messages describing public health research findings to inform their decision-making. We also sought to examine differences in preferences between public health policy-makers and practitioners.

**Methods:**

A cross sectional, value-weighting survey of policy-makers and practitioners was conducted. Participants were asked to allocate a proportion of 100 points across different (i) sources of research evidence, (ii) message content and (iii) the form in which evidence is presented. Points were allocated based on their rating of influence, usefulness and preference when making decisions about health policy or practice.

**Results:**

A total of 186 survey responses were received from 90 policy-makers and 96 practitioners. Researchers and government department agencies were the most influential source of research evidence based on mean allocation of points, followed by knowledge brokers, professional peers and associations. Mean point allocation for perceived usefulness of message content was highest for simple summary of key findings and implications, and then evidence-based recommendations and data and statistical summaries. Finally, based on mean scores, policy-makers and practitioners preferred to receive research evidence in the form of peer-reviewed publications, reports, evidence briefs and plain language summaries. There were few differences in scores between policy-makers and practitioners across source, message content or form assessments or those with experience in different behavioural areas.

**Conclusions:**

The findings should provide a basis for the future development and optimization of dissemination strategies to this important stakeholder group.

**Supplementary Information:**

The online version contains supplementary material available at 10.1186/s12961-023-01066-7.

## Background

Evidence is a powerful tool for public health improvement and its use is a core element of prudent public health decision-making [1]. However, many government-funded public health programmes and services are not well supported by evidence [2]. Studies undertaken in the the United States of America (USA) and Europe, for example, suggest only 50–65% of public health policies and programmes are evidence based [3, 4]. Strategies to increase the use of evidence in decision-making are required to improve the impact of research on public health polices and services.

Knowledge translation (KT) is the process in which research informs policy and practice to improve community health. Specifically it is defined as “The synthesis, exchange, and application of knowledge by relevant stakeholders to accelerate the benefits of global and local innovation in strengthening health systems and improving people’s health”. [5] Initially, strategies to facilitate knowledge translation focused on improving access and availability of evidence to public health policy-makers and practitioners, and then, to improve their capacity to find, appraise and apply research to their decision-making [6]. More contemporary approaches reflect the complexity of the process, and suggest it is better achieved through comprehensive strategies including research coproduction and knowledge exchange processes between knowledge producers (for example, researchers) and users (for example, public health policy-makers and practitioners) [7].

The dissemination of research is an important component of comprehensive knowledge translation strategies. Dissemination is defined by Rabin et al. as “an active approach of spreading evidence-based interventions to the target audience via determined forms using planned strategies” [8]. It may be a particularly useful strategy to reach public health policy-makers or practitioners from agencies or sectors not engaged in research co-production but who may have an interest in the research findings. Public health researchers engage in range of dissemination strategies. An international study across the United Kingdom of Great Britain and Northern Ireland (UK), the USA and Brazil, for example, found 39% of public health researchers used media interviews and 12% targeted mailing to disseminate their research [9]. Other surveys of health researchers, primarily from the USA, found health researchers typically issued press releases (33%), newsletters (36%), policy briefs (21%), used social media (42%) and conducted face-to-face meetings with stakeholders (55%) to disseminate research findings [10].

Understanding preferences of policy-makers and practitioners for receiving research evidence is important to support research use in decision-making. Brownson’s model for dissemination of research describes factors that may influence the salience and potential impact of dissemination strategies on the decision-making of public health policy-makers and practitioners [11]. These include the message source (from whom is the research being disseminated), the content (characteristics and clarity of the message and so on) and the form or modality in which it was disseminated. Relatively little research, however, has been undertaken to describe how such factors could best be tailored to public health policy-makers and practitioners. Surveys of policy-makers in the UK report research derived from local data, qualitative research and systematic reviews were particularly useful to their decision-making [12]. The study also reported research from other government departments, review articles, recognized experts and professional associations were frequently used sources of evidence [12]. Research with European public health decision-makers found the presentation of research findings in a way that clearly communicated key messages, its relevance to practice and the inclusion of actionable recommendations may be particularly helpful to support evidence use [13]. Similarly, studies describing different forms of dissemination have found seminars or workshops (59%) followed by academic journals (50%), email alerts (40%) and policy briefs (30%) were preferred by state health department officials to learn about public health research [14].

Previous research has largely relied on interviews or instruments that assess the frequency of public health policy-makers or practitioner ratings of the utility or preference of dissemination strategies [15, 16]. While useful, these measures provide limited capacity to differentiate between strategies based on the extent to which they are preferred or valued. Value-weighting methods provide an opportunity to quantify the relative preference or value of different dissemination strategies from the perspective of public health policy-makers or practitioners [17] by allocating a finite number of points across a range of potential options. Research using such techniques to systematically examine each of the components of the model for dissemination of research (that is, the source, content and form of messages) would provide new insights to support the design of dissemination strategies which may be more effective in communicating research findings to policy-makers and practitioners.

The aim of this study was to quantify and describe the views of public health policy-makers and practitioners regarding the source, content and form of messages describing public health research findings. We also sought to examine differences in preferences between public health policy-makers and practitioners and area of expertise.

## Methods

### Design and setting

An online cross-sectional value-weighting survey was conducted from May to October 2021 with Australian-based public health prevention policy-makers and practitioners. The survey was developed by the research team and underwent an iterative process of development with feedback from an expert panel which included members of the Australian Prevention Partnership Centre and the Local Health District, prior to pilot testing with three policy-makers/practitioners. Ethics approval was provided by the University of Newcastle Human Research Ethics Committee (H-2014-0070). Implied consent was obtained. Participants were not provided any form of incentive for their participation in this study.

### Participants

Participants were eligible if they had worked as an Australian public health prevention policy-maker or practitioner at a government or nongovernment health organization within the past 5 years. Policy-makers were defined as a person who makes decisions, plans and actions that are undertaken to achieve specific public health prevention goals on behalf of a government or nongovernment organization [18]. Practitioners were defined as a person actively engaged in the delivery of public health prevention programmes, implementing services or models of care in health and community settings (definition developed by research team).

#### Recruitment

Leaders of government (for example, local health promotion units, government departments of health) and nongovernment (for example, the Cancer Council) organizations, professional associations (for example, the Public Health Association of Australia) and research–practice partnerships (for example, the Australian Prevention Partnership Centre) were identified through Google searches, were emailed invitations to participate in the study, and were asked to distribute to appropriate staff or through their networks. Australian practitioners registered with the International Union for Health Promotion and Education (IUHPE) were contacted and sent an invitation to participate via public domain emails or on LinkedIn (where identified by the research team). Authors who had published public health related articles between 2018 and 2021 in the Australian and New Zealand Journal of Public Health (ANZJPH), Health Promotion Journal of Australia (HPJA) and Public Health Research and Practice (PHRP), and had listed an affiliation with a public health policy or practice organization were also sent direct emails inviting participation. Finally, opportunities to participate were also promoted on the social media account of the National Centre of Implementation Science on Twitter and LinkedIn.

The invitation emails included: links to the ethics-approved information statement, where participants were informed about the purpose of the study and that the findings would be published; and a link to the online survey. Reminder emails were sent to nonresponders at approximately 2 and 4 weeks following the initial email invitation. The reminder emails contained the same links (that is, information statements and online survey) provided in the initial invitation email.

### Data collection and measures

The online survey was administered using the REDCap software [19], a secure web-based application for building and managing online surveys and databases. Upon commencing the survey, a unique nonidentifying code was automatically generated to allow each participant to access and re-enter the survey if they were unable to complete it in one sitting. The survey took approximately 15–20 minutes to complete.

#### Demographics

Participants were asked to indicate if they had been a policy-maker or practitioner in the past 5 years. If employed in more than one role, participants were asked to identify their primary role. Participants also completed items assessing the Australian state or territory where they work as a policy-makers or practitioner; whether their employer was a government, nongovernment (not for profit), or industry (for profit) organization; how long they had worked in the field of public health policy or practice; if they had completed a PhD; and if they had professional experience in any of the following public health prevention fields: nutrition and dietetics; physical activity or sedentary behaviour; overweight or obesity; tobacco, alcohol or other drugs; sexual health; oral health; injury prevention; violence prevention; mental health; or infectious diseases. These categories were not mutually exclusive.

#### Source, content and form

Participants completed a value-weighting exercise separately, to quantify their views regarding the usefulness of different attributes of source, content and form for receiving public health research evidence. For each question, participants were asked to allocate a proportion of 100 points across options (see Additional file 1 for a full list of options presented). Lists of attributes were developed based on previous qualitative and quantitative research [9, 10, 12–15, 20]. A higher allocation of points represented a greater level of importance participants perceived for an attribute. Items that were not allocated any points were assumed to represent a rating of zero.

Source: Participants were asked “Please indicate how influential receipt of evidence from the following sources (from whom) would be to your decision-making as a public health policy-maker or practitioner. Please allocate your 100 points across as many (or as few) of the sources in the table below. Allocate more points to sources you think are more influential. Allocate a zero (‘0’) or leave blank those sources that would not have a meaningful influence in your decision-making. The total points allocated across all sources must equal 100”.

Content: “How useful to your decision-making as a public health policy-maker or practitioner is the following content (what is received) within a document, presentation or other evidence delivery format. Please allocate your 100 points across as many (or as few) content elements in the table below. Allocate more points to content you think are more useful. Any content that is not useful for your decision-making leave blank or allocate a zero (‘0’). The total points allocated across all content elements must equal 100”.

Form: “In what form would you prefer to receive evidence to inform a policy or practice decision. Please allocate your 100 points across as many forms (forms to receive evidence) in the table below. Allocate more points to content you more strongly preference. Forms that are not preferred leave blank or allocate a zero (‘0’). The total points allocated across all content elements must equal 100”.

### Statistical analysis

Data were managed and the analysis was undertaken in SAS v 9.3 software. We used descriptive statistics to describe the sample characteristics and findings of participant preferences. Similar to other value-weighting studies [17], we calculated the mean allocation of points for each item across the three outcomes (that is, source, content and form) and ranked these scores in ascending order to allow for identification of the most to least preferred items. As allocation of points used a free response survey field, in instances where participants allocated more or less than 100 points, their allocation of points were standardized to 100. This was calculated overall for policy-makers and practitioners combined as well as separately by role. Differences in the allocation of points were compared between the two roles using *t*-tests. The mean allocation of points were also calculated by the participants reported expertise (for example, nutrition, physical activity, mental health). This was to allow for descriptive exploration of how preferences may vary by area of expertise. Due to the small sample sizes and the lack of independence between content areas, as participants could select experience in more than one area, statistical comparisons were not made, and results are reported in subgroups with samples > 30 participants. Where response options were missing, these were not included in the results and the denominator for the item was adjusted accordingly.

## Results

Two hundred ninety-two survey responses were received (255 via the email invite and 37 via the social media platform). Of these, 106 were ineligible for the following reasons: participant role was unknown (*n* = 53), did not identify as a public health policy-maker or practitioner (*n* = 32), duplicate entries (*n* = 20) and located outside of Australia (*n* = 1). As a result, 186 participants were eligible (Table 1) of these individuals, 143, 141 and 141 completed the source, content and form survey questions, respectively. Response options for the remaining individuals were missing.

**Table 1:** Participant characteristics

Characteristic^a^	Total (*n* = 186)	Policy-maker (*n* = 90)	Practitioner (*n* = 95)
Holds a PhD qualification	63 (34%)	29 (32%)	34 (36%)
Length of time working in public health			
< 5 years	34 (18%)	10 (11%)	24 (25%)
5–15 years	83 (45%)	38 (42%)	45 (47%)
15+ years	68 (37%)	42 (47%)	26 (27%)
Experience in public health topic^b^			
Overweight or obesity	90 (48%)	44 (49%)	46 (48%)
Nutrition and dietetics	85 (46%)	34 (38%)	51 (53%)
Physical activity or sedentary behaviour	83 (45%)	41 (46%)	42 (44%)
Tobacco, alcohol or other drugs	79 (42%)	46 (51%)	33 (34%)
Mental health	53 (28%)	33 (37%)	20 (21%)
Sexual health	35 (19%)	22 (24%)	13 (14%)
Infectious diseases	33 (18%)	20 (22%)	13 (14%)
Injury prevention	31 (17%)	21 (23%)	10 (10%)
Oral health	16 (8.6%)	9 (10%)	7 (7.3%)
Violence prevention	13 (7.0%)	9 (10%)	4 (4.2%)

^a^Cell totals may not add to equal total sample size due to missing values

^b^Cells do not add to equal 100% as participants could select more than one area of experience

### Perceived influence of the source of messages

**Fig. 1:**
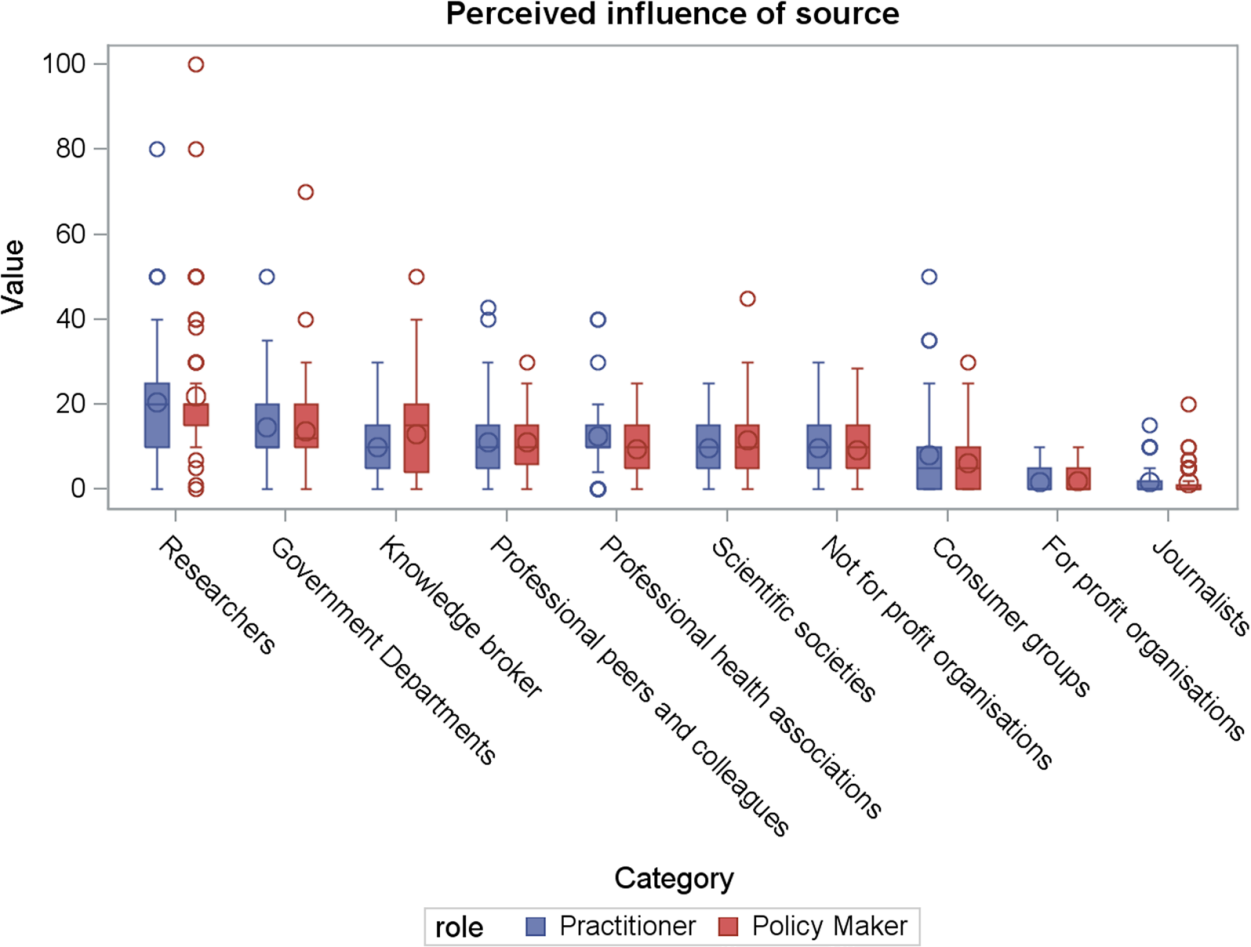
Box plot of the perceived influence of the source of research evidence split by role

One hundred forty-three participants (*n* = 72 policy-makers, *n* = 71 practitioners, 43 missing) completed items assessing the perceived influence of receipt of evidence from difference sources on public health policy and practice decisions (Table 2, Fig. 1). For policy-makers and practitioners combined, allocation of points was greatest for “researchers”, indicating they were perceived as most influential source of evidence (mean = 21, SD = 14.5), followed by “government departments” (mean = 14, SD = 10.0). Allocation of points for the next highest, namely knowledge brokers, influential professional peers and public health associations were similar, ranging from 11 to 12. For-profit organizations and journalists were the least influential source of research evidence, with mean scores of 2.

**Table 2:** Perceived influence of the source of research evidence

Response option	Policy-makers (*n* = 72)		Practitioners (*n* = 71)		Estimate 95% CI	All (*n* = 143)	
	Mean (SD)	Rank	Mean (SD)	Rank		Mean (SD)	Rank
Researchers (for example, those who undertook the research or those with whom you have an existing professional relationship)	22 (15.6)	1	21 (13.4)	1	1.62 (− 3.56, 6.07)	21 (14.5)	1
Government departments or agencies (for example, Department of Health)	14 (10.3)	2	15 (9.7)	2	−0.88 (−4.19, 2.44)	14 (10.0)	2
Knowledge broker, that is, an individual or organization with both research and policy practice expertise that facilitates the transfer and exchange of information	13 (10.6)	3	10 (7.5)	5	3.03 (−0.01, 6.06)	12 (9.3)	3
Influential professional peers and colleagues (for example, opinion leader)	11 (6.9)	5	11 (8.3)	4	0.14 (−2.38, 2.66)	11 (7.6)	4
Professional health associations (for example, Public Health Association of Australia, Australian Medical Association)	10 (6.2)	6	13 (7.9)	3	−3.13 (−5.47, −0.80)	11 (7.2)^a^	5
Scientific societies or bodies (for example, the Society for Behavioural Medicine)	12 (8.1)	4	10 (6.7)	6	1.91 (−0.54, 4.37)	11 (7.5)	6
Nongovernment, not for profit organizations (foundations, charities, for example, the Cancer Council, the Heart Foundation)	9 (6.4)	7	10 (6.3)	7	−0.42 (−2.52, 1.67)	9 (6.3)	7
Consumer groups or relevant individual patients, consumers, community members(for example, the Consumer Health Forum of Australia)	6 (6.1)	8	8 (9.7)	8	−2.08 (−4.74, 0.59)	7 (8.1)	8
Nongovernment, for-profit organizations or agencies operating on their behalf (for example, commercial industry)	2 (3.1)	9	2 (3.0)	9	0.22 (−0.79, 1.24)	2 (3.1)	9
Journalists (for example, the news media)	2 (3.3)	10	2 (3.2)	10	−0.05 (−1.13, 1.03)	2 (3.3)	10

^a^Difference in scores allocated by policy-makers and practitioners based on results from *t*-tests

Professional associations were allocated fewer points on average by policy-makers compared with practitioners, otherwise there were no other statistically significant differences in point allocation between the two groups (*P* = 0.0191).

Mean scores and ranks were also similar between those with expertise across a range of content areas, including those with expertise in nutrition and dietetics; physical activity or sedentary behaviour; overweight or obesity; tobacco, alcohol or other drugs; and mental health (Additional file 1).

### Perceived usefulness of message content

**Fig. 2:**
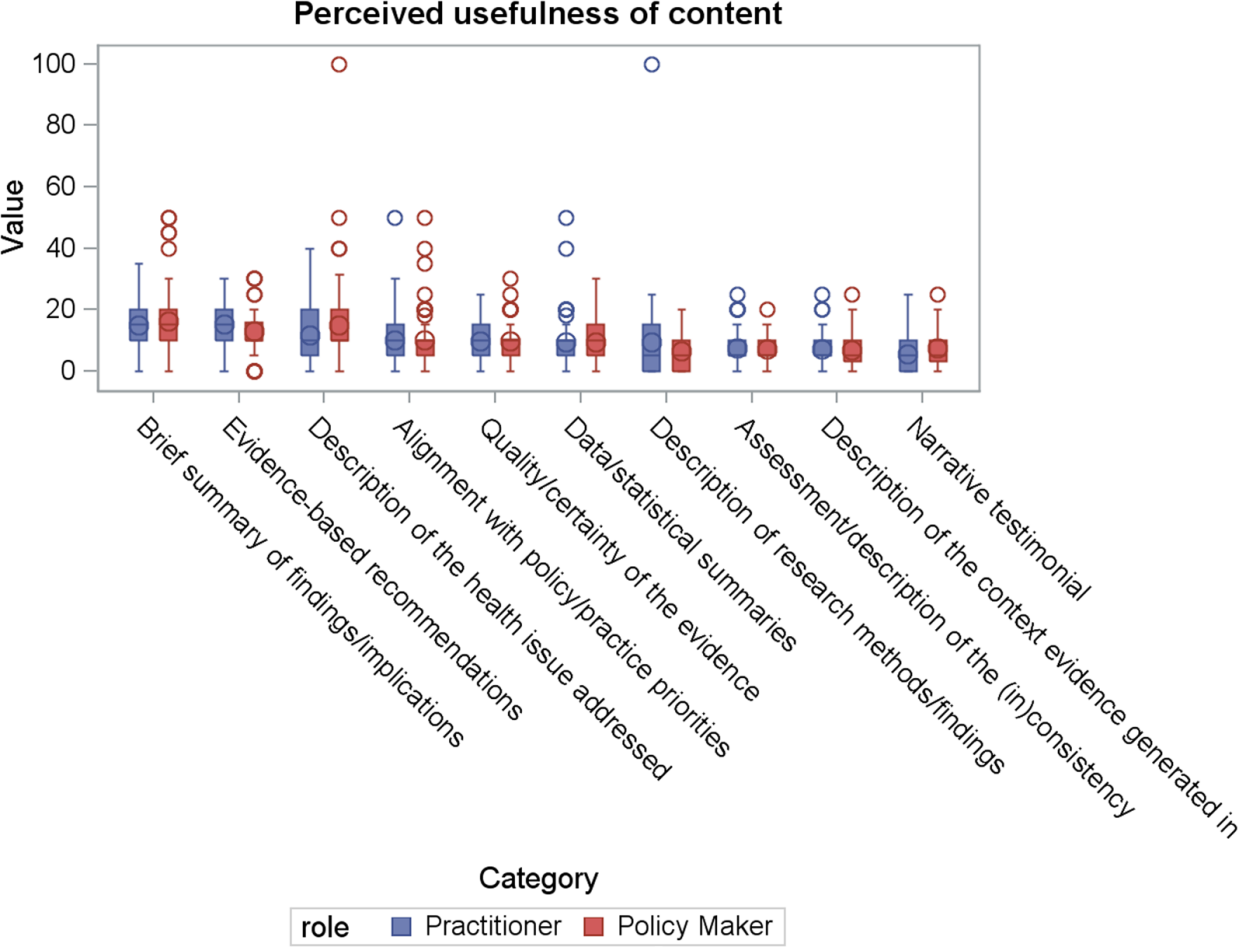
Box plot of the perceived usefulness of message content of research evidence split by role

One hundred forty-one participants (*n* = 71 policy-makers, *n* = 70 practitioners, 45 missing) answered the value-weighting questions indicating their perceived usefulness of different message content to inform their public health policy and practice. For policy-makers and practitioners combined, allocation of points was highest for dissemination strategies that included a simple summary of the key findings and implications (mean = 16, SD = 9.7). The inclusion of evidence-based recommendations (mean = 141, SD = 7.3), and data and statistical summaries or presentations (mean = 13, SD = 11.8) were ranked second and third preferred message content attributes, respectively. This was followed by a grouping of three message attributes where mean scores were similar, namely a description of alignment of the research evidence with a local policy or practice priority, assessment of the quality or certainty of the evidence, and a description of the health issue the research sought to address (range mean = 9 to 10). The use of narrative, story or testimonial was ranked lowest, though still accrued an average point allocation score of 6 (Table 3, Fig. 2).

**Table 3:** Perceived usefulness of message content

Response option	Policy-makers (*n* = 71)		Practitioners (*n* = 70)		Estimate 95% CI	All (*n* = 141)	
	Mean (SD)	Rank	Mean (SD)	Rank		Mean (SD)	Rank
A brief simple summary of the research, key findings and implications	16 (11.0)	1	15 (8.2)	2	1.29 (−1.95, 4.52)	16 (9.7)	1
Evidence-based recommendations regarding a future course of action	13 (7.2)	3	15 (7.2)	1	−2.41 (−4.80, −0.02)	14 (7.3)^a^	2
Data and statistical summaries or presentations of the evidence to describe the impact of a health issue or intervention	15 (14.2)	2	12 (8.5)	3	3.16 (−0.75, 7.08)	13 (11.8)	3
A description of the alignment of the research with local policy or practice priorities	10 (9.2)	4	10 (8.2)	4	0.06 (−2.85, 2.96)	10 (8.7)	4
Assessments regarding the quality or certainty of the evidence	10 (6.3)	5	10 (6.5)	5	−0.28 (−2.40, 1.85)	10 (6.4)	5
A description of the health issue or problem the research sought to address	9 (7.3)	6	9 (8.4)	7	0.10 (−2.53, 2.73)	9 (7.9)	6
A complete and detailed description of research methods and findings	6 (5.7)	10	9 (13.2)	6	−2.92 (−6.30, 0.46)	8 (10.2)	7
An assessment or description of the (in)consistency of the research findings with the broader scientific literature	7 (4.6)	8	7 (6.0)	8	−0.15 (−1.92, 1.63)	7 (5.3)	8
An assessment or description of the context in which the evidence was generated	7 (5.4)	9	7 (5.3)	9	−0.60 (−2.36, 1.17)	7 (5.3)	9
The use of narrative, story or testimonial to describe the impact of a health issue or intervention	7 (6.4)	7	6 (5.7)	10	1.75 (−0.26, 3.75)	6 (6.1)	10

^a^Difference in scores allocated by policy-makers and practitioners based on results from *t*-tests

The inclusion of evidence-based recommendations was scored lower by policy-makers compared with practitioners, (*P* = 0.0362), otherwise there were no other statistically significant differences for any other options between these two groups. Mean scores and ranks for the top most useful attributes were also broadly similar between those with expertise across a range of content areas, including those with expertise in nutrition and dietetics; physical activity or sedentary behaviour; overweight or obesity; tobacco, alcohol or other drugs; and mental health (Additional file 1).

### Preferences for the form of messages

**Fig. 3:**
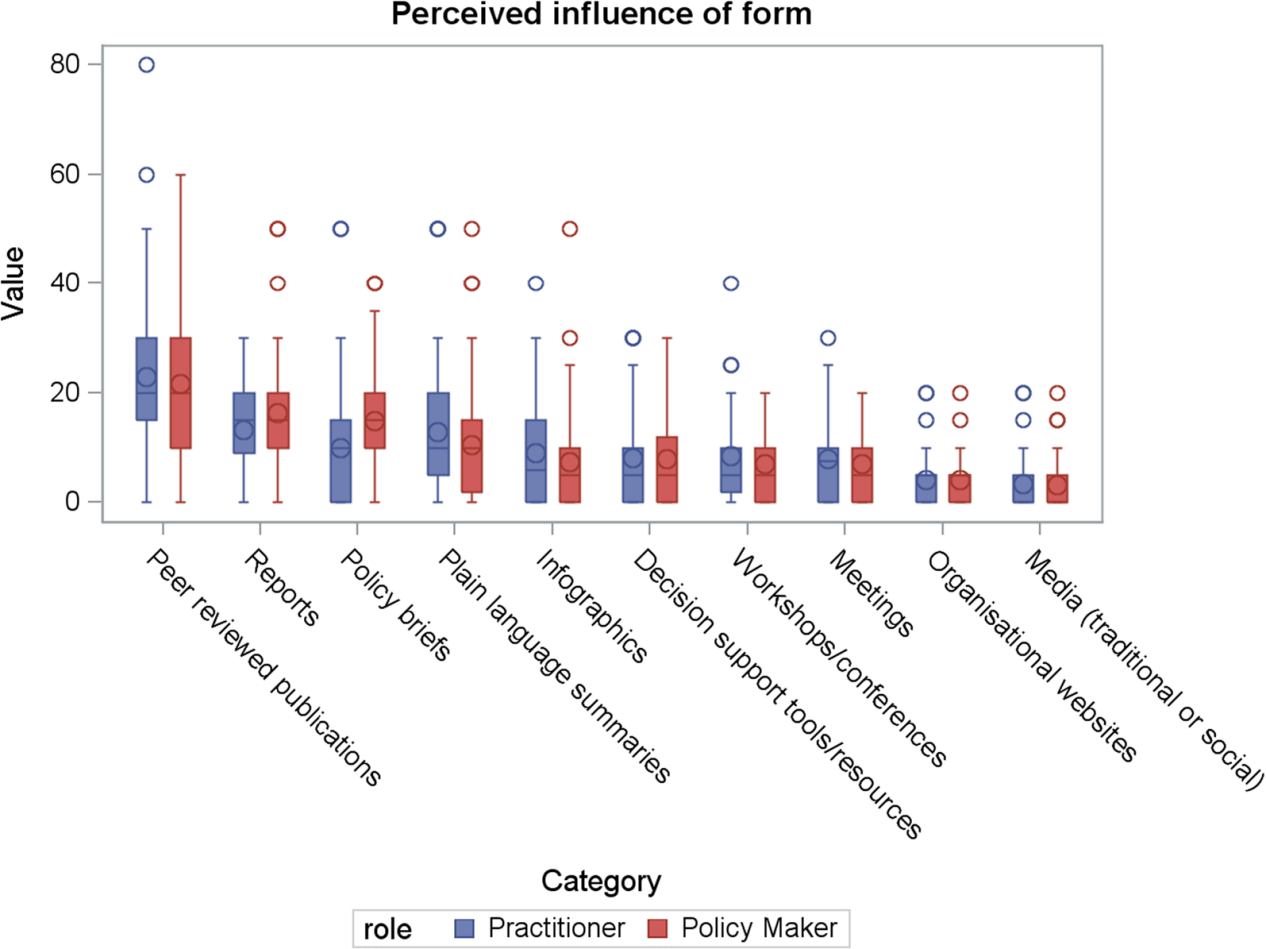
Box plot of the perceived influence of the form of research evidence split by role

One hundred forty-one participants (*n* = 71 policy-makers, *n* = 70 practitioners, 45 missing) answered the value-weighting questions indicating their perceived usefulness of the form of messages to inform their public health policy and practice. Mean allocation of points regarding the preferred form to received public health research evidence for both policy-makers and practitioners combined was greatest for peer-reviewed publications (mean = 22, SD = 15.1). This was followed by reports (mean = 15, SD = 9.3), policy briefs (mean = 12, SD = 9.9) and plain language summaries (mean = 12, SD = 11.2). The provision of information on organizational websites and media (traditional or social) were the least preferred. There was little difference in scores between the other proposed forms for the provision of research evidence (Table 4, Fig. 3).

**Table 4:** Perceived influence of the form of research evidence

Response option	Policy-makers (*n* = 71)		Practitioners (*n* = 70)		Estimate 95% CI	All (*n* = 141)	
	Mean (SD)	Rank	Mean (SD)	Rank		Mean (SD)	Rank
Peer-reviewed publications	22 (15.1)	1	23 (15.1)	1	−1.29 (−6.33, 3.74)	22 (15.1)	1
Reports	16 (9.6)	2	13 (8.8)	2	3.00 (−0.06, 6.05)	15 (9.3)	2
Policy briefs	15 (9.4)	3	10 (10.0)	4	5.00 (1.77, 8.20)	12 (9.9)^a^	3
Plain language summaries	10 (10.3)	4	13 (12.1)	3	−2.39 (−6.13, 1.34)	12 (11.2)	4
Infographics	7 (8.9)	6	9 (9.1)	5	−1.55 (−4.53, 1.43)	8 (9.0)	5
Decision support tools or resources	8 (7.7)	5	8 (8.2)	7	−0.12 (−2.78, 2.52)	8 (7.9)	6
Workshops or conferences	7 (5.8)	8	9 (8.1)	6	−1.44 (−3.79, 0.90)	8 (7.0)	7
Meetings (in person or technology enabled)	7 (5.8)	7	8 (7.5)	8	−0.87 (−3.10, 1.36)	8 (6.7)	8
Organizational websites	4 (4.4)	9	4 (4.7)	9	−0.03 (−1.55, 1.49)	4 (4.5)	9
Media (traditional or social)	3 (4.4)	10	3 (4.7)	10	−0.27 (−1.78, 1.23)	3 (4.5)	10

^a^Difference in scores allocated by policy-makers and practitioners based on the results from *t*-tests

Policy briefs were preferred to a greater extent by policy-makers compared with practitioners (*P* < 0.001), otherwise there were no other statistically significant differences in the allocated scores between these two groups. Mean scores for the top-ranking attributes were also broadly similar between those with expertise across different content areas (Additional file 1).

## Discussion

This study examined the views of Australian public health policy-makers and practitioners regarding the source, content and form they may receive public health research evidence to inform their decision-making. The study found that policy-makers and practitioners perceive evidence disseminated by researchers and government health departments as most influential and messages that are brief, have clear recommendations for action, include data summaries and a description of its alignment with local policy or practice priorities as most useful. The study also found practitioners and policy-makers preferred research distributed via peer-reviewed publications, reports, policy briefs or plain language summaries. There was broad similarity in views of policy-makers and practitioners, and between those with expertise across a range of public health priority areas. Given the considerable scope to improve the salience and use of evidence in public health decision-making, such findings are important in that they offer some guidance on how this could be achieved [12].

There has been relatively little research describing the perceived influence of difference sources of research evidence on health policy. Prior work has suggested the vested interests and actions of industry groups (such as alcohol or tobacco) [21] may undermine their perceived credibility as a source of research evidence. While some studies have also identified vested interests among researchers [22] and government agencies [23]. The findings of this study indicate public health policy-makers and practitioners clearly perceive researchers as the most influential source of health evidence. The findings suggest that researchers and the academic institutions should feature prominently in research dissemination efforts targeting public health policy and practice.

Regarding message content, policy-makers and practitioners perceived brief summaries and evidence-based recommendations regarding future courses of action as most useful to support decision-making. Such findings are consistent with qualitative research of public health policy-makers and practitioners in North America, who suggest they have a need for research that concisely communicate clear key messages and policy guidance [24]. Findings also support current initiatives of many academic journals, including Cochrane, whereby full-text publications, specifically of systematic reviews, are accompanied by key messages regarding implications for policy and practice [13]. While preferred, brief summaries may provide limited opportunity to communicate complex research findings that may require nuanced or elaborate explanation. In such circumstances, other more interactive forms of engagement with policy-makers and practitioners may be required [25]. The inclusion of data summaries and presentations, ranked third in this study, is also consistent with research suggesting public health policy-makers and practitioners value data in decision-making, and underscores the importance of data visualization strategies as a means of communicating research findings [26].

Interestingly, policy-makers and practitioners preferred academic publications as a dissemination form. The findings underscore the importance of open access methods of publication to enable research to be freely available and accessible to policy-makers and practitioners globally [27]. Other forms for research dissemination that provide greater opportunity for interaction, such as conferences and workshops, were not rated highly by study participants. Some research suggests such opportunities provide particularly useful forums for knowledge exchange, with surveys of state health department staff in the USA indicating seminars or workshops (59%) were preferred over academic journals (50%) and policy briefs (30%) as a means of learning about public health research [14]. Potentially, the apparent discordance of this findings with past research may reflect contextual differences in how policy-makers and practitioners use research. In this study, participants were asked to report the preference of forms or research evidence for instrumental/symbolic use – that is, to directly inform (or justify) a policy or practice decision. Static forms of evidence (for example, reports) may be more useful for such evidence use [28]. However, more intensive and interactive workshops or seminars may be preferred for conceptual use of research, that is, its use for understanding or enlightenment regarding a priority issue [28]. Further research to determine if and how preferences change based on the purpose for research use, and across the policy-making process is warranted and may enable more sophisticated targeting of dissemination strategies.

There were relatively few differences in the average scores across source, content or form attributes between policy-makers and practitioners. Previous studies have also shown similar use of evidence among these groups [28]. Perhaps understandably, policy-makers had a stronger preference for policy briefs as a form for receiving research evidence. This is likely a reflection of the potential familiarity policy-makers have with such documents and their direct tailoring of this format to their needs [29]. Practitioners had a greater preference for professional associations as a source of evidence, and for the inclusion of evidence-based recommendations as part of research dissemination strategies. This finding may be attributable, in part, to the role of professional associations in guiding clinical and public health practice. For example, explicit recommendations regarding “best practice” are often provided to practitioners from professional association via guidelines [30]. Nonetheless, given many health policies are without a strong evidence basis [3, 4], further research to understand how to enhance the salience of such information for decision-making among policy-makers is warranted.

This study used a convenience sample of Australian-based policy-makers and practitioners. The generalizability of these findings to policy-makers and practitioners in other jurisdictions, therefore, may be limited. Furthermore, the findings may have been influenced by selection bias. While the random selection of policy-makers and practitioners from across jurisdictions may address these limitations, we were unaware of such databases to sample from, either in Australia or internationally. The survey items were also not validated. Future research establishing the psychometric properties of the items is therefore warranted. Finally, we sought to characterize differences in preferences between policy-makers and practitioners. While a number of differences were found, the size of the study sample provided limited power to do so. As such, small but meaningful differences between groups may not have been identified.

## Conclusions

Research has identified effective policies and practices capable of improving public health exist across a range settings [31–34]. Evidence to inform their population-wide implementation is also accruing [35–39]. A range of factors influence the use of evidence in health policy and practice decision-making [40]. The dissemination strategies represent one approach to overcome some of the barriers to evidence use. This study quantified the extent to which public health policy-makers and practitioners’ value attributes of the source, content and form in which research evidence is distributed to them. The study found broad alignment between the attributes valued by policy-makers and practitioners. It also found they consider evidence disseminated by researchers most influential; message content which is brief and have clear recommendations for action as most useful; and prefer research distributed via peer-reviewed publications, reports, policy briefs or plain language summaries. A range of dissemination source, message and form attributes were rated highly by participants, suggesting that comprehensive dissemination approaches that use a range of strategies are most likely to be effective. The findings should provide a basis for the future development and optimization [41] of dissemination strategies to this important stakeholder group.

## Contribution to the literature

- Research has identified effective public health policies and practices across a range of settings.
- Dissemination and application of this research is necessary to maximize its potential impacts.
- This study found policy-makers and practitioners value evidence disseminated by researchers, brief and clear recommendations, and peer-reviewed publications.
- Using these findings will be helpful when disseminating research to policy-makers and practitioners.

### Supplementary Information

**Additional file 1: Table S1.** Perceived influence of the source of research evidence by topic area. **Figure S1.** Line graph representing mean points allocated for sources of information, overall and by topic area. **Table S2.** Perceived influence of message content by topic area. **Figure S2.** Line graph representing mean points allocated for message content, overall and by topic area. **Table S3.** Perceived influence of research evidence form by topic area. **Figure S3.** Line graph representing mean points allocated for message form, overall and by topic area.

## Data Availability

The datasets used and/or analysed during the current study are available from the corresponding author on reasonable request.

## Abbreviations

ANZJPH: Australian and New Zealand Journal of Public Health
HPJA: Health Promotion Journal of Australia
IUHPE: International Union for Health Promotion and Education
KT: Knowledge translation
PHRP: Public health research and practice
SD: Standard deviation
UK: United Kingdom
USA: United States of America
