# Supplementary material for: Disseminating health research to public health policy-makers and practitioners: a survey of source, message content and delivery modality preferences
Source: Health Res Policy Syst. 2023 Nov 27;21:121. doi: 10.1186/s12961-023-01066-7 (PMC10680334; doi:10.1186/s12961-023-01066-7)
Supplement: Supplementary file 1 — Additional file 1: Table S1. Perceived influence of the source of research evidence by topic area. Figure S1. Line graph representing mean points allocated for sources of information, overall and by topic area. Table S2. Perceived influence of message content by topic area. Figure S2. Line graph representing mean points allocated for message content, overall and by topic area. Table S3. Perceived influence of research evidence form by topic area. Figure S3. Line graph representing mean points allocated for message form, overall and by topic area. [file 12961_2023_1066_MOESM1_ESM.docx]

| **Source** | **Overall (n=143)** | **Nutrition and dietetics (n=68)** | **Physical activity or Sedentary behaviour (n=64)** | **Overweight or obesity (n=72)** | **Tobacco, Alcohol or Other drugs (n=57)** | **Mental health (n=41)** |
| --- | --- | --- | --- | --- | --- | --- |
| **Researchers (e.g. those whom undertook the research or those with whom you have an existing professional relationship)** | 21.2 | 19.29 | 19.16 | 18.07 | 19.21 | 19.44 |
| **Government Departments or Agencies (e.g. Department of Health)** | 14.18 | 14.7 | 14.97 | 15.11 | 15.42 | 14.05 |
| **Knowledge broker i.e.an individual or organisation with both research and policy practice expertise that facilitates the transfer and exchange of information** | 11.5 | 10.98 | 10.96 | 11.7 | 11.69 | 11.7 |
| **Influential professional peers and colleagues (e.g. opinion leader)** | 11.15 | 12.6 | 12.73 | 13.26 | 11.86 | 9.32 |
| **Professional health associations (e.g. Public Health Association of Australia, Australian Medical Association)** | 11.07 | 11.95 | 11.3 | 10.56 | 10.59 | 10.74 |
| **Scientific societies or bodies (e.g. the Society for Behavioural Medicine)** | 10.72 | 10.05 | 10.23 | 10.14 | 11.06 | 12.01 |
| **Non-government, not for profit organisations (Foundations, Charities, e.g. Cancer Council, Heart Foundation)** | 9.45 | 10.32 | 9.39 | 10 | 10.49 | 9.04 |
| **Consumer groups or relevant individual patients, consumers, community members(e.g. Consumer Health Forum of Australia)** | 7.15 | 6.96 | 7.3 | 7.11 | 5.94 | 9 |
| **Non-government, for profit organisations or agencies operating on their behalf (e.g. commercial industry)** | 1.93 | 1.75 | 2.13 | 2.25 | 1.84 | 2.39 |
| **Journalists (e.g. the news media)** | 1.65 | 1.4 | 1.84 | 1.79 | 1.89 | 2.32 |

| **Content** | **Overall (n=141)** | **Nutrition and dietetics (n=67)** | **Physical activity or Sedentary behaviour (n=62)** | **Overweight or obesity (n=71)** | **Tobacco, Alcohol or Other drugs (n=56)** | **Mental health (n=41)** |
| --- | --- | --- | --- | --- | --- | --- |
| **A brief simple summary of the research, key findings and implications.** | 15.55 | 16.71 | 15.68 | 15.91 | 15.43 | 14.96 |
| **Evidence based recommendations regarding a future course of action.** | 13.91 | 14.62 | 14.51 | 13.87 | 12.76 | 12.2 |
| **Data and statistical summaries or presentations of the evidence to describe the impact of a health issue or intervention.** | 13.26 | 11.13 | 10.71 | 11.2 | 15.21 | 13.24 |
| **A description of the alignment of the research with local policy or practice priorities.** | 10.03 | 10.93 | 10.28 | 10.83 | 9.74 | 9.01 |
| **Assessments regarding the quality or certainty of the evidence.** | 9.64 | 9.82 | 8.99 | 9.35 | 10.48 | 9.75 |
| **A description of the health issue or problem the research sought to address.** | 9.28 | 8.62 | 10.05 | 9.59 | 7.98 | 9.53 |
| **A complete and detailed description of research methods and findings.** | 7.89 | 7.13 | 7.3 | 7.01 | 6.99 | 7.57 |
| **An assessment or description of the (in)consistency of the research findings with the broader scientific literature.** | 7.17 | 7.08 | 7.09 | 7.09 | 7.85 | 7.65 |
| **An assessment or description of the context in which the evidence was generated.** | 6.83 | 6.96 | 7.09 | 7.49 | 7.13 | 8.77 |
| **The use of narrative, story or testimonial to describe the impact of a health issue or intervention.** | 6.44 | 6.99 | 8.3 | 7.67 | 6.43 | 7.33 |

| **Formats** | **Overall (n=141)** | **Nutrition and dietetics (n=67)** | **Physical activity or Sedentary behaviour (n=61)** | **Overweight or obesity (n=70)** | **Tobacco, Alcohol or Other drugs (n=56)** | **Mental health (n=41)** |
| --- | --- | --- | --- | --- | --- | --- |
| **Peer reviewed publications** | 22.28 | 19.87 | 19.48 | 19.66 | 24.05 | 22.02 |
| **Reports** | 14.78 | 12.69 | 13.48 | 13.73 | 15.29 | 15.76 |
| **Policy briefs** | 12.4 | 11.67 | 13.67 | 14.54 | 14.82 | 12.66 |
| **Plain language summaries** | 11.67 | 12.63 | 12.21 | 11.09 | 9.86 | 11.02 |
| **Infographics** | 8.19 | 9.18 | 7.95 | 7.67 | 7.27 | 8.05 |
| **Decision support tools or resources** | 7.98 | 9.3 | 9.49 | 8.61 | 6.46 | 7.61 |
| **Workshops or conferences** | 7.8 | 9.52 | 8.57 | 8.23 | 7.64 | 7.2 |
| **Meetings (in person or technology enabled)** | 7.52 | 8.06 | 8.41 | 9.33 | 6.3 | 7.07 |
| **Organisational websites** | 4.07 | 3.69 | 3.61 | 3.6 | 4.46 | 5.12 |
| **Media (traditional or social)** | 3.32 | 3.4 | 3.13 | 3.54 | 3.84 | 3.49 |
